# Recent advances in (chemo-)radiation therapy for rectal cancer: a comprehensive review

**DOI:** 10.1186/s13014-020-01695-0

**Published:** 2020-11-10

**Authors:** F. Roeder, E. Meldolesi, S. Gerum, V. Valentini, C. Rödel

**Affiliations:** 1 Department of Radiotherapy and Radiation Oncology, Paracelsus Medical University, Landeskrankenhaus, Müllner Hautpstrasse 48, 5020 Salzburg, Austria; 2 Dipartimento di Diagnostica per Immagini, Radioterapia Oncologica ed Ematologia, UOC Radioterapia Oncologica, Fondazione Policlinico Universitario “A. Gemelli” IRCCS, Roma, Italy; 3 Department of Radiotherapy, University of Frankfurt, Frankfurt, Germany

**Keywords:** Rectal cancer, Chemoradiation, Review

## Abstract

The role of radiation therapy in the treatment of (colo)-rectal cancer has changed dramatically over the past decades. Introduced with the aim of reducing the high rates of local recurrences after conventional surgery, major developments in imaging, surgical technique, systemic therapy and radiation delivery have now created a much more complex environment leading to a more personalized approach. Functional aspects including reduction of acute or late treatment-related side effects, sphincter or even organ-preservation and the unsolved problem of still high distant failure rates have become more important while local recurrence rates can be kept low in the vast majority of patients. This review summarizes the actual role of radiation therapy in different subgroups of patients with rectal cancer, including the current standard approach in different subgroups as well as recent developments focusing on neoadjuvant treatment intensification and/or non-operative treatment approaches aiming at organ-preservation.

## Introduction and current standard approach

Rectal cancer represents approximately one-third of all colorectal cancer with the second highest incidence and the second highest cause of cancer death in the western society [1]. Considering the restricted role of radiotherapy in the treatment of colon disease, we’ll focus our review mostly on rectal cancer where radiotherapy has a leading position in combination with both surgery and chemotherapy.

During the last 3 decades, the role of radiation therapy in the management of locally advanced rectal cancers, has been gradually modified. Starting in the ‘80 s with a prevalent adjuvant role due to its potential in reducing pelvic recurrence after surgical resection and increasing survival rates when combined with 5-FU based chemotherapy [2], radiotherapy was challenged, in the early ‘90 s, with the introduction of total mesorectal excision (TME) that significantly decreased locoregional recurrence (LRR) by itself, questioning the necessity of radiotherapy before or after surgery [3]. Several short course (5 Gy × 5 days) randomized trials [4–9] have demonstrated the importance of preoperative RT plus TME in reducing LRR, in stage II and III rectal cancer patients. The assumption that adding chemotherapy to long course (45–50 Gy) preoperative radiotherapy could increase the local effect of radiotherapy, led to the comparison between radiotherapy and radiochemotherapy as neoadjuvant regimen [10]. The addition of concomitant chemotherapy to preoperative radiotherapy resulted in a significant increase in local control while only slightly increasing acute toxicity, without affecting adherence to radiotherapy, feasibility of surgery (with no increase of postoperative morbitidy), or adherence to adjuvant chemotherapy. However, no significant improvement in overall survival was observed in any single trial.

Over the time, the availability of different treatment options (including radiotherapy and chemotherapy) and the possibility to use different regimens (pre- and postoperative), has resulted in an increasing demand of reliable preoperative staging. Different imaging techniques have been used to locally stage rectal cancer with variable sensitivities and specificities [11]. High-resolution MRI has been shown to be superior to clinical examination, computer tomography and endoluminal ultrasound (EUS) for rectal cancer staging [12]. The possibility to have more accurate information related to the pelvic structures as the possibility to distinguish a tumor from rectal wall, to depict the mesorectal fascia [13], to identify anatomical structures useful to support an optimal surgical technique [14] and to better characterize suspicious lymph nodes [15], made MRI the principal imaging technique in the assessment of a rectal cancer. Based on MRI imaging, able to identify poor prognostic factors preoperatively, it was possible to divide rectal cancer patients into three groups (“good”, “bad” and “ugly”), according to their local and systemic failure’s risks [16].

For “good” tumors, surgery alone is the mainstay of treatment. Only for tumors located in the distal rectum, radiochemotherapy can be considered with a neo-adjuvant/definitive intent to increase either sphincter preservation or achieve organ preservation by omission of surgery or a combination with local excision in selected cases.

Considering patients having “bad” MRI features, neoadjuvant treatment has been established to reduce both the risks of LLR and distant metastases. Two different regimens have been tested in those patients: conventional long-course radiochemotherapy (LCRT: 45–50 Gy with 1.8–2 Gy fractions over 5–6 weeks), mostly used in South Europe and in the United States and short-course radiotherapy (SCRT: 5 Gy × 5 fractions) without preoperative chemotherapy, mostly used in the North of Europe. Several studies [7–9, 17, 18] have investigated the two regimens in the past, even if the enrolled population was not completely comparable considering that the SCRT regimens included patients with early tumor (stage T1–T2 and some resectable T3), while LCRT studies considered mainly more locally advanced rectal cancer patients (T3, T4 and unresectable tumors). Although LCRT was expected to have advantages of higher sphincter preservation and lower complication rates, several phase III randomized studies [19, 20] have found no difference in oncological outcomes (DFS, OS, local relapse-free survival). However, LCRT schedule showed higher pathological complete response (pCR) rate and clear resection margin. Similar results were obtained in the study of Ngan et al. [21] with a trend of better local control rate of LCRT in distal rectal lesions. Stating that there is not a real evidence to recommend one treatment modality over the other, the results of Ngan et al. justify the common practice to treat a tumor that is located in the distal rectum, close to the anal sphincter and/or locally advanced cT4 or CRM-positive tumors with a LCRT schedule.

In order to test if the longer interval between the end of radiotherapy and surgery was responsible for the higher pCR rate of the LC schedule, a SCRT regimen with delayed surgery (between 6 and 8 weeks after radiotherapy) was tested. A higher pCR rate was reported for delayed as compared with immediate surgery after SCRT [22]. Similar oncological outcomes were observed for early and delayed SCRT, with a higher acute radiation toxicity rate in the delayed and a significantly higher rate of postoperative complications in the early surgery group.

Considering the essential improvement in LC reached with the modern neoadjuvant treatments and surgical TME technique, the reduction of treatment-related side-effects and postoperative complications is now a priority. In LCRT and SCRT with delay, a 6–8 weeks break after RT is considered standard, with a higher surgical morbidity when surgery is delayed for 11 weeks after LCRT according to a randomized trial [23], although this finding could not be confirmed in other prospective trials [24, 25]

For the “ugly” group, characterized by a high risk for local recurrence and distant metastasis, neoadjuvant LCRT is recommended. To optimize the treatment, systemic chemotherapy can be administered either before or after neoadjuvant LCRT/SCRT, referred to as Total neoadjuvant therapy (TNT) [26].

Finally, the benefit of adjuvant chemotherapy in patients with locally advanced rectal cancer treated with neoadjuvant SCRT or LCRT is still highly controversial [27].

Although RT is accepted as an essential component of multidisciplinary treatment (MDT), specific issues still remain unaddressed. It’s well defined that neoadjuvant LCRT followed by TME surgery is recommended for locally advanced rectal cancer, but what about early rectal cancer located in the lower rectum? And more, what is the role of the TNT and the chemotherapy intensification in rectal cancer? This review will recall the controversial issues and analyze the recent advances in the radiation therapy field.

## Neoadjuvant treatment intensification

During the last decades, an increasing interest in intensified treatment has been paid, mainly focused on locally advanced and metastatic rectal cancer. Using the standard chemoradiation approach, only about 11–18% of patients will achieve a pathological complete remission (pCR) [28–33]. Because this small group of patients shows a clearly improved overall prognosis compared to patients with less or no response [34], several strategies have been explored to improve the pCR rate or even omit surgery in selected cases. Those include the use of more intensive chemotherapy regimens concurrent to radiation, addition of targeting agents to concurrent chemoradiation, escalation of radiation dose or the use of altered fractionations, and the sequential use of (chemo)radiation and (intensified) induction or consolidation chemotherapy regimes in the neoadjuvant setting (total neoadjuvant therapy, TNT).

The highest level of evidence exists for adding oxaliplatin to standard 5-FU based neoadjuvant chemoradiation. Results from six phase III trials ([28–33, 35, 36], Table 1) addressing this issue have been published so far. Although two reported significantly increased pCR rates [28, 33] and one significantly improved disease-free-survival (DFS) [28], all others failed to show any significant improvements in major oncological endpoints while reporting increased toxicities [29–32, 35]. Taken the slightly different treatment schedules into account, there might be a (small) benefit for the addition of (dose-dense) oxaliplatin, however its efficacy does not seem to be high enough as a sole strategy. Nevertheless, adding oxaliplatin to concurrent chemoradiation might be still considered in patients with urgent need to downsizing or embedded in TNT- or non-operative management (NOM)-approaches.

**Table 1:** Phase III trials of neoadjuvant concurrent chemoradiation approaches with the addition of oxaliplatin

Trial	Phase	Disease stage	n	Ind. CHT	RT dose^4^	Conc. CHT	Adj. CHT	pCR rate	downstaging	3y-DFS	grade 3+ tox
ARO 2004 [28]	III	T3-4 or N+	1236	None	50.4	5-FU	5-FU	**13%**	n.r	**71%**	20%
				None	50.4	5-FU/Ox	5-FU/LV/Ox	**17%**	n.r	**76%**	24%
NSABP-R04 [29]^1^	III	T3-4 or N1-2	1608	None	50.4–55.8	5-FU or Cap	n.s	18%	n.r	n.r	**28%**
				None	50.4–55.8	5-FU or Cap+ Ox	n.s	20%	n.r	n.r	**41%**
ACCORD 12 [30, 35]	III	T3-4 or T2 dist	598	None	45	Cap	n.s	14%	29%^4^	68%	**11%**
				None	50	Cap/Ox	n.s	19%	39%^4^	73%	**25%**
STAR-1 [31]	III	T3-4 or N1-2	747	None	50.4	5-FU	5-FU	16%	n.r	n.r	**8%**
				None	50.4	5-FU/Ox	5-FU	16%	n.r	n.r	**24%**
PETACC-6 [32]^2^	III	T3-4 or N+	1094	None	45–50.4	Cap	Cap	11%	44%^5^	74%	**15%**
				None	45–50.4	Cap/Ox	Cap/Ox	13%	42%^5^	75%	**37%**
FOWARC [33, 36]^3^	III	T3-4 or N1-2	330	5-FU/LV	46–50.4	5-FU/LV	5-FU/LV	**14%**	**39%**^**6**^	73%	Tox sig. inc
				mFOLFOX6	46–50.4	mFOLFOX6	mFOLFOX6	**28%**	**60%**^**6**^	77%	With Ox

n: number of patients, RT dose: radiation therapy dose in Gy, conc. CHT: concurrent chemotherapy, ind. CHT: induction chemotherapy (prior to RT), adj. CHT: adjuvant chemotherapy (after surgery), pCR rate: percentage of patients with pathological complete remission, downstaging: percentage of patients with major downstaging defined according to study protocol (only listed if combined T and N downstaging was reported), 3y-DFS: 3 year rate of disease free survival, grade3+ tox: acute toxicity during combined chemoradiation grade 3 or higher (only listed if an overall percentage was reported), dist.: distal, 1: 2 × 2 factorial design after amendment, 2: published as abstract only, 3: three arm design, only arms with radiation therapy reported, 4: defined as DWORAK score 2 and 3, 5: defined as ypT0-2N0, 6: defined as ypstage 0–1, 5-FU: 5-fluorouracil, Ox: Oxaliplatin, Cap: capecitabine, LV: leucovorin, mFOLFOX6: combination regimen of 5-fluorouracil, leucovorin and oxaliplatin, tox sig. inc.: toxicity significantly increased, bold style: indicates significant difference

Irinotecan was also tested early as an adjunct to standard 5-FU based chemoradiation based on its known activity in metastatic colorectal cancer [37]. Several phase I-II trials and one phase III trial ([38–56], Table 2) evaluating different dose schedules have reported conflicting results with pCR rates of 10–38% (weighted average 24%). While the only phase III trial showed a significantly improved pCR rate at the cost of increased toxicity [55], the only randomized phase II trial failed to show any benefit of the addition of irinotecan to standard 5-FU based chemoradiation [51, 52]. Moreover, another randomized phase II trial comparing the addition of Irinotecan or Oxaliplatin to standard chemoradiation reported a pCR rate of only 12% with Irinotecan but 23% with Oxaliplatin with similar toxicity [49, 50]. There is some evidence that patients with certain UGT1A1 genotypes may respond better to irinotecan-based therapies [53–55], which may allow a better patient selection in the future. Until then, the results from the ongoing British phase III ARISTOTLE trial are awaited.

**Table 2:** Phase I/II trials of neoadjuvant concurrent chemoradiation approaches with the addition of irinotecan

Author	Year	Phase	Disease stage	n	RT dose	Conc. CHT	Adj. CHT	pCR rate (%)	downst	3y-DFS	tox gr.3+	postop. c
Mehta [38]	2003	II	T3 or T2N+	32	50.4	5-FU ci + Iri	n.s	38	71%^1^	n.r	n.r	n.r
Hofheinz [39]	2005	I	T3/4	19	50.4	Cap (esc) + Iri	n.s	21	75%^1^	n.r	DLT 21%	11%^20^
Klautke [40]	2005	II	T3/4 or T2N+ ^2^	37	50.4	5-FU ci + Iri	5-FU +/− FA	22	n.r	73%^17^	n.r	n.r
Klautke [41]	2006	I/II	T3/4 or T2N+	28	55.8	Cap (esc) + Iri	5-FU + / − FA	16	n.r	n.r	20%^19^	4%
Navarro [42]	2006	II	T3/4	74	45	5-FU ci + Iri	dis	14	49% ^1^	n.r	n.r	n.r
Willeke [43]	2007	II	T3/4 or N+	36	50	Cap + Iri	dis	15	n.r	n.r	DLT 19%	n.r
Glynne-Jones [44]	2007	I/II	T3/4^3^	57	45	5-FU/LV + Iri (esc)	n.s	21	41%^1^	n.r	DLT 12%	n.r
Shin [45]	2010	II	T3/4 or N+	43	50.4	S-1 + Iri	n.s	21	n.r	72%	23%	n.r
Gollins [46]	2011	I/II	T3/4^4^	110	45	Cap + Iri	dis	22	n.r	64%	n.r	n.r
Sato [47]	2011	II	T3/4 or N+	67	45	S-1 + Iri	S-1 + Iri^12^	35	n.r	n.r	15%	3%^20^
Hong [48]	2011	II	T3/4	48	50.4	Cap + Iri	Cap	25	n.r	75%^18^	no gr. 4	5%^20^
Wong [49, 50]	2012	II, rand	T3/4	111^5^	50.4	Cap + Iri	FOLFOX	12	n.r	68%^17^	27%	19%
RTOG 0247					50.4	Cap + Ox	FOLFOX	23	n.r	62%^17^	27%	20%
Mohiuddin [51, 52]	2013	II, rand	T3/4	106	55.2–60^13^	5-FU	rec.^6^	28	n.r	n.r	42%	n.r
RTOG 0012					50.4–54	5-FU + Iri	rec.^6^	28	n.r	n.r	51%	n.r
Zhu [53]	2018	I	T3-4^7^	26	50	Cap + Iri (esc)^14^	n.s	25	n.r	n.r	DLT 20%	4%^20^
Guan [54]	2019	II	T3/4^8^	52	50	Cap + Iri^14^	Cap +/− Ox^21^	28	n.r	n.r	38%	n.r
Zhang [55]^9^	2019	III	T3/4^7^	360	50	Cap^15^	n.r	**18**	n.r	n.r	Tox sig	n. sig
CinClare					50	Cap + Iri^10,14^	n.r	**34**	n.r	n.r	inc. w. Iri	
Wang [56]	2020	Pooled	T3/4 or N+	371	50	Cap + Iri (var)^14^	XELOX^16^	23^11^	n.r	n.r	n.r	n.r

n = number of patients, RT dose: radiation dose in Gy, conc. CHT: concurrent chemotherapy, adj. CHT: adjuvant chemotherapy (after surgery), pCR rate: percentage of patients with pathological complete remission, downst.: percentage of patients with major downstaging defined according to study protocol (only listed if combined T and N downstaging was reported), 3y-DFS: 3-year disease-free survival, tox gr.3+: acute toxicity during combined chemoradiation grade 3 or higher (only listed if an overall percentage was reported), postop. c.: postoperative complications grade 3 or higher (only listed if an overall percentage was reported), 5-FU: 5-Fluorouracil, ci: contineous infusion, Iri: Irinotecan, mg: milligram, n.s.: not specified, n.r.: not reported, Cap: capecitabine, esc: doses were escalated during study, FA: folinic acid, y: years, dis.: at discretion of the treating physician, LV: leucovorin, S-1: oral fluoropyrimidin prodrug, DLT: dose limiting toxicity as specified in the protocol, Ox: Oxaliplatin, rand.: randomized, FOLFOX: combination regimen including 5-Fluorouracil, Leucovorin and Oxaliplatin, RTOG: radiation therapy oncology group, XELIRI: combination regimen including capecitabine and Irinotecan, XELOX: combination regimen including capecitabine and Oxaliplatin, sig. significantly, inc.: increased, w.: with, gr.: grade, mFOLFOX6: FOLFOX: combination regimen including 5-Fluorouracil, Leucovorin and Oxaliplatin as specified in the protocol, 1: downstaging defined as reduction from clinical to pathological stage, 2: uncertain R0 resection or sphincter preservation, 3: fixed tumors or threated mesorectal fascia, 4: tumor threatening (≤ 2 mm) or exceeding mesorectal fascia, 5: only patients after study amendment evaluated, 6: adjuvant chemotherapy was recommended for all patients with residual disease, in patients without residual disease Chemotherapy was at the discretion of the treating physician, 7: limited to patients with UGT1A1*1*1 or *1*28 phenotype, 8: limited to patients with UGTA1*1*1 phenotype, 9: published as abstract only, 10: Irinotecan dose was based on UGTA1 phenotype, var: variable doses, 11: pCR rate significantly increaesd with > 4 cycles of Irinotecan, 12: in patients with ypN+, 13: hyperfractionated RT with 1.2 Gy 2 times per day, 14: one cycle consolidation chemotherapy (prior to surgery) with XELIRI, 15: one cycle of consolidation chemotherapy (prior to surgery) with XELOX, 16: mFOLFOX6 also allowed, 17: 4-year rates, 18: 5-year rates, 19: in phase II part, 20: re-operation rate, n. sig.: no signficant difference between arms, 21: 5-FU instead of Cap allowed, bold style: indicates significant difference

Bevacizumab, a monoclonal antibody targeting vascular epithelial growth factor (VEGF), is part of most current standard first-line multidrug regimens used in metastatic colorectal cancer [57, 58]. Therefore it has been evaluated as an adjunct to 5-FU or 5-FU/Oxaliplatin based chemoradiation in numerous phase I and II studies for rectal cancer ([59–77], Table 3). Reported pCR rates range from 8 to 40% with a weighted average of 19%. The only randomized phase II trial [75] found a small but significant benefit in terms of pCR compared to standard chemoradiation with no significant increase in acute side effects or postoperative morbidity. However, several others have reported high rates of severe acute toxicities and postoperative complications mainly in terms of impaired or delayed wound healing [64, 69] which led to delayed or omitted adjuvant chemotherapy in a high percentage of patients. Therefore Bevacizumab does not seem to be an ideal candidate for treatment intensification at least in neoadjuvant settings outside a NOM approach.

**Table 3:** Phase I/II trials of neoadjuvant concurrent chemoradiation approaches with the addition of bevacizumab

Author	Year	Phase	Disease stage	n	RT dose	Conc. CHT	Adj. CHT	pCR rate (%)	Downst	Tox gr.3+	Postop. c
Crane [59]	2010	II	T3 N0-1	25	50.4	Cap + Bev	rec	32	84%^7^	4%	12%^9^
Willett [60]	2010	I/II	T3/4	32	50.4	5-FU ci + Bev	rec	16	n.r	n.r	n.r
Kourkourakis [61]	2010	II	T3 or N+	19	34 4	Cap + Bev + Ami	Cap	37	n.r	n.r	5%
Liang [62]	2011	II	T3/4 or N+	28	45	FOLFOX + Bev	n.s	25	n.r	n.r	n.r
Velenik [63]	2011	II	T3/4 or N+	61	50.4	Cap + Bev	Cap	13	74%^7^	n.r	10%^9^
Di Petrillo [64]	2012	II	T3/4 or N+	26	50.4	5-FU ci + Ox + Bev^5^	rec	20	n.r	76%	n.r
Gasparini [65]	2012	II	T3/4 or T2N+	43	50.4	Cap + Bev	5-FU/LV ± Bev	14	n.r	n.r	8%
Resch [66]	2012	II	T3	8	45	Cap + Bev	dis	25	n.r	50%^8^	25%
Kennecke [67]	2012	II	T3/4 or N2	42	50.4	Cap + Ox + Bev	n.s	18	n.r	n.r	11%^9^
Spigel [68]^1^	2012	II	T3/4 or N+	35	50.4	5-FU + Bev	FOLFOX + Bev	29	n.r	n.r	n.r
Landry [69]	2013	II	T3/4	53	50.4	Cap + Ox + Bev	FOLFOX + Bev	17	59%^7^	72%	6%^9,10^
Dellas [70]	2013	II	T3/4^3^	70	50.4	Cap + Ox + Bev	Cap	17	n.r	11%	16%
Wang [71]	2014	II	T3/4 or N+	12	45	FOLFOX + Bev	n.s	33	n.r	25%	0%
Borg [72]^2^	2014	II	T3	45	45	5-FU ci + Bev	n.s	11	n.r	20%	20%
Garcia [73]	2015	II	T3/4	43	45	Cap + Bev	Cap or CAPOX^11^	8	78%^7^	n.r	n.r
Sadahiro [74]	2015	II	T3/4 or N+	52	45	S-1 + Bev	S-1	19	71%^7^	2%	29%
Salazar [75]	2015	II, rand	T3/4 or N+	90	45	Cap + Bev	n.s	**16**	73%^7^	16%	16%
					45	Cap		**11**	78%^7^	13%	7%
Maeda [76]	2018	II	T3/4 or N+	25	50.4	Cap + Bev	Cap	16	n.r	0%	n.r
Yu [77]	2018	II	T3/4 or N+	45	50	Cap + Ox + Bev^6^	XELOX → Cap	40	n.r	20%	13%^9^

n: number of patients, RT dose: radiation dose in Gy, conc. CHT: concurent chemotherapy, adj. CHT: adjuvant chemotherpy, pCR rate: percentage ofpatients with pathologic complete remission, downst.: percentage of patients with major downstaging defined according to study protocol (only listed if combined T and N downstaging was reported), tox gr. 3+: acute grade 3+ toxicity during chemoradiation (only listed if an overall percentage was reported), postop. c.: postoperative complications grade 3+ (only listed if an overall percentage was reported), rand.: randomized, 5-FU: 5-fluorouracil, ci: contineous infusion, Ox: Oxaliplatin, Bev: Bevacizumab, Cap: Capecitabine, Ami: amifostine, FOLFOX: combination regimen including 5-fluorouracil, leucovorin and oxaliplatin, XELOX: combination regimen including capecitabine and oxaliplatin, S-1: oral fluoropyrimidin prodrug, LV: leucovorine, CAPOX: combination regimen including Capecitabine and Oxaliplatin, rec.: recommended, dis.: at the discretion of the treating physician, n.s.: not specified, n.r.: not reported, 1: only data from preoperative chemoradiation arm included, 2: only data from study arm without induction chemotherapy included, 3: included 17% patients with M1, 4: in 10 fractions, 5: 1 cycle of FOLFOX + Bev as induction chemotherapy, 6: 1 cycle of XELOX + Bev as induction chemotherapy, 7: downstaging defined as reduction in pathological stage compared to clinical stage, 8: study terminated due to predefined toxicity criteria, 9: complications requiring surgical intervention, 10: late surgical complications of any grade in 47%, 11: Cap for ypN0, CAPOX or FOLFOX for ypN+ patients, bold style indicates significant difference

Cetuximab, a monoclonal antibody targeting the Epidermal growth factor (EGFR), is part of the current standard multidrug regimen in metastatic KRAS wild-type colorectal cancer [57, 58]. Therefore it (as well as other anti-EGRF antibodies like Panitumumab or downstream tyrosinkinase inhibitors like Gefitinib) has been evaluated in combination with standard 5-FU based-chemoradiation in various phase I and II trials ([78–95], Table 4) also for rectal cancer. Although most of them showed a modest toxicity profile, results in terms of pCR rates were mainly disappointing (pCR rates 0–27%, weighted average 14%). Two randomized phase II trials comparing Capecitabine- and CAPOX-based chemoradiation with or without anti-EGFR agents did not observe a significant benefit for their addition [88, 89]. Moreover, neither KRAS status nor EGFR-expression seems a robust predictor of pCR [88].

**Table 4:** Phase I/II trials of neoadjuvant concurrent chemoradiation approaches with the addition of EGFR-pathway targeting agents

Author	Year	Phase	KRAS	Disease stage	n	RT dose	Conc. CHT	Adj. CHT	pCR rate (%)	downst	tox gr.3+	postop. c
Machiels [78]	2007	I/II	n.s	T3/4 or N+	40	45	Cap + Cet	dis	5	38%^7^	n.r	13%
Roedel [79]	2008	I/II	n.s	T3/4 or N+	60	50.4	Cap + Ox + Cet	n.s	9	n.r	n.r	11%^9^
Valentini [80]	2008	I/II	n.s	T3 or T2N+	41	50.4	5-FU ci + Gef	5-FU/LV^11^	27	73%^8^	41%	0%
Bertolini [81]	2009	II	n.s	T3/4	40	50–50.4	5-FU ci + Cet^3^	n.s	8	45%^8^	n.r	n.r
Horisberger [82]	2009	II	n.s	T3/4 or N+	50	50.4	Cap + Iri + Cet	n.s	8	n.r	n.r	n.r
Velenik [83]	2010	II	n.s	T3/4 or N+	37	45	Cap + Cet^4^	Cap	8	73%^7^	n.r	5%^9^
Kim [84]	2011	II	n.s	T3/4 or N+	40	50.4	Cap + Iri + Cet	5-FU/LV	23	n.r	18%	5%
Pinto [85]	2011	II	n.s	T3N+ or T4	60	50.4	5-FU ci + Ox + Pan	FOLFOX + Pan	21	58%^7^	n.r	n.r
Sun [86]	2012	II	n.s	T3/4 or N+	63	45	Cap + Cet	dis	13	78%^7^	n.r	n.r
Kim [87]	2012	Pooled	wt	T3/4 or N+	62	50.4	Cap + Iri	5-FU/LV	21	44%^8^	n.r	n.r
							Cap + Iri + Cet	5-FU/LV	28	56%^8^	n.r	n.r
Dewdney [88]	2012	II, rand	n.s	T3c/T4^1^	165	50.4	Cap^5^	CAPOX	15	n.r	n.r	n.r
Expert-C						50.4	Cap + Cet^6^	CAPOX + Cet	18	n.r	n.r	n.r
Helbling [89]	2013	II, rand	wt	T3/4 or N+	40	45	Cap + Pan	rec	10	n.r	n.r	18%^10^
SAKK 41/07					28	45	Cap		18	n.r	n.r	15%^10^
Eisterer [90]	2014	II	n.s	T3/4	31	45	Cap + Cet	n.s	0	n.r	n.r	n.r
Mardjuadi [91]	2015	II	wt	T3/4 or N+	19	45	Pan	n.s	0	n.r	n.r	n.r
Jin [92]	2015	II	n.s	T3/4 or N+	23	50.4	Cap + Nim^3^	CAPOX	19	n.r	n.r	n.r
Bazarbashi [93]	2016	II	n.s	T3/4 or N+	15	50.4	Cap + Cet	5-FU/LV or Cap	13	n.r	n.r	n.r
Gollins [94]	2017	II	n.s	MRF+ or dis.^2^	82	45	Cap + Iri + Cet	dis	17	49%^7^	59%	n.r
Pinto [95]	2018	II	wt	T3 or T2N+	98	50.4	Pan	FOLFOX	11	46%^7^	n.r	n.r

KRAS: KRAS status of included patients, n: number of patients, RT dose: radiation dose in Gray, conc. CHT: concurrent chemotherapy, adj. CHT: adjuvant chemotherapy, pCR rate: percentage of patients with complete pathologic remission, downst.: percentage of patients with major downstaging according to study protocol (only listed if combined T and N downstaging was reported), tox gr.3+: acute Grade 3+ toxicity during chemoradiation (only listed if an overall percentage was reported), postop. c.: postoperative complications grade 3+ (only listed if an overall percentage was reported), rand.: randomized, n.s.: not specified, wt: wild type, MRF: mesorectal fascia, dis.: distal tumors, Cap: Capecitabine, Cet: Cetuximab, Ox: Oxaliplatin, 5-FU: 5-fluorouracil, ci: contineous infusion, Gef: gefitinib, Iri: Irinotecan, Pan: Panitumumab, Nim: Nimotizumab, CAPOX: combination regimen including capecitabine and oxaliplatin, dis: at the discretion of the treating physician, LV: leucovorine, FOLFOX: combination regimen including 5-FU, leucovorine and oxaliplatin, rec. recommended, bold style indicates significant difference, 1: or threatened mesorectal fascia (< 1 mm) or EVSI or tumor at levator level, 2: MRF+ defined as tumor < 1 mm of mesorectal fascia or involved fascia, 3: 3 times cetuximab mono as induction, 4: 2 weeks capecitabine mono as induction, 5: 4 cycles CAPOX as induction, 6: 4 cycles CAPOX + Cetuximab as induction, 7: downstaging defined as reduction of pathological stage versus clinical stage, 8: downstaging defined as yp stage 0–1, 9: re-operation rate, 10: rate of surgical interventions, 11: if ypN+

Several other agents have been tested in phase I or II trials concurrent to chemoradiation based on more or less robust preclinical and/or clinical evidence either for their activity in colorectal cancer or for enhancing radiation effects. Those include classic chemotherapy agents like Cisplatin [96], Mitomycin C [97], or Temozolomide [98], COX-2-inhibitors like Celecoxib [99–101], proteasome inhibitors like Bortezomib [102], PI3K-akt-inhibitors like Nelfinavir [103], phosphatidylserine-antibodies like Bavituximab [104], Multi-Tyrosine-Kinase-Inhibitors like Sorafenib [105, 106], PARP-Inhibitors like Veliparib [107] and fusion proteins like Aflibercept [108] (listed in Table 5). Reported pCR rates varied from 7 to 39%. Although the addition of some agents resulted in promising pCR rates with acceptable toxicities, these findings should be interpreted as preliminary and further research is warranted.

**Table 5:** Phase I/II trials with neoadjuvant concurrent chemoradiation approaches with the addition of other substances

Author	Year	Phase	Disease stage	n	RT dose	Conc. CHT	Adj. CHT	pCR rate (%)	Downst	Tox gr.3+	Postop. c
Valentini [96]	2008	IIb	T3	164	50.4	Cis/5-FU	dis.^4^	22^6^	52%^7^	7%	10%^10^
					50.4	Ox + Ralti	dis.^4^	28^6^	58%^7^	16%	6%^10^
Stojanovic [97]	2011	II	T3/4 or N+	49	45	Cap + MMC	5-FU/LV	16	27%^7^	n.r	16%
Jeong [98]	2016	I	T3/4 or N+	22	50.4	Cap + Tem (esc)	n.s	32^5^	n.r	18%	n.r
Jakobsen [99]	2008	II	T3/4	35	60^2^	UFT + Cele	n.s	21	n.r	6% (49%^9^)	n.r
Debucquoy [100]	2009	II, rand	T3/4 or N+	35	45	5-FU ci + Cele	dis	39^6^	n.r	0%	n.r
						5-FU ci		29^6^	n.r	3%	n.r
Wang [101]	2014	II	T3/4 or N+	53	44	UFT + FA + Cele	FOLFOX	13	n.r	6% (gr. 4)	n.r
O´Neil [102]	2010	I	T3/4 or N+	10	50.4	5-FU ci + Borte (esc)	n.s	10	40%^7^	DLT 40%	n.r
Buijsen [103]	2013	I	T3/4 or N+	11	50.4	Cap + Nel (esc)	n.s	27	82%^7^	55%	9%
Meyer [104]	2018	I	T3/4 or N+	14	50.4	Cap + Bavi (esc)	dis	7	64%^8^	25%	21%
von Moos [105]	2018	I/II	T3/4 or N+^1^	54	45	Cap + Sor (esc)	rec	15	n.r	n.r	13%
Kim [106]	2016	I	T3/4 or N+	17	50.4	5-FU ci + Sor (esc)	n.s	33	87%^8^	18%	n.r
Czito [107]	2017	Ib	T3/4 or N+	32	50.4	Cap + Veli (esc)	rec	29	71%^7^	25%	n.r
Bendell [108]	2017	II	T3/4 or N+	39	50.4	5-FU + Afli	FOLFOX + Afli	23	n.r	n.r	11%

n = number of patients, RT dose: radiation dose in Gy, conc. CHT: concurrent chemotherapy, adj. CHT: adjuvant chemotherapy, pCR rate: percentage of patients with pathologic complete regression, downstaging: percentage of patients with major downstaging defined according to study protocol (only listed if combined T and N downstaging was reported), tox gr.3+: acute grade 3+ toxicity during chemoradiation (only listed if an overall percentage was reported), postop. c.: postoperative complications grade 3+ (only listed if an overall percentage was reported), rand.: randomized, Cap: Capecitabine, MMC: Mitomycin C, esc: dose escalated, 5-FU: 5-fluorouracil, ci: contineous infusion, UFT: uracil-tegafur, FA: folinic acid, Cis: Cisplatin, Ox: Oxaliplatin, CAPOX: combination regimen including capecitabine and oxaliplatin, LV: leukovorin, Tem: Temozolomide, Cele: Celecoxib, Ralti: Raltitrexed, Borte: Bortezomib, Nel: Nelfinavir, Bavi: Bavituximab, Sor: Sorafenib, Veli: Veliparib, Afli: Aflibercept, n.s.: not specified, dis.: at the discretion of the treating physician, FOLFOX: combination regimen including 5-fluorouracil, leucovorine and oxaliplatin, rec.: recommended, n.r.: not reported, gr.: grade, DLT: dose limiting toxicity as predefined in the protocol, bold style: indicates significant difference, 1: only KRAS mutant patients included, 2: patients received an additional brachytherapy boost with 5 Gy in 1 fraction, 3: patients recived one consolidation cycle of CAPOX prior to surgery, 4: recommended only for ypN+ patients, 5: significantly increased pCR rate in n = 16 patients with methylated MGMT promotor compared to n = 6 patients without MGMT methylation (38% vs 17%), 6: no significant difference, 7: downstaging defined as yp stage 0–1, 8: downstaging defined as reduction of yp stage compared to c stage, 9: treatment with celecoxib finished due to rash (although all grade 1–2), 10: anastomotic leakage

Another possibility of improving chemoradiation effects is simply to increase radiation dose. Since Appelt et al. [109] provided clear evidence for a dose–response relationship between 50,4 and 70 Gy dependent on pretreatment T- and N-category [109], various prospective observational and phase I/II trial have evaluated dose escalation in the mentioned range within different concurrent chemotherapy regimens ([51, 52, 99, 110–142], Table 6). Dose escalation was achieved by either adding more fractions in conventional fractionation, using altered fractionation regimes or by adding a brachytherapy boost. Reported pCR rates ranged from 0 to 50% with a weighted average of 22% (excluding the population based trial [137]). Mohiuddin et al. [51, 52] reported a comparative study using conventional fractionation to either standard dose (45–50 Gy) or escalated dose (55–60 Gy) and observed a significantly increased pCR rate with dose escalation (13% vs 44%). Regarding brachytherapy boosts, one phase II trial with a matched cohort found a significant increase in pCR rates (12% vs 29%) [134] while a Danish phase III trial did not observe a significant difference in pCR rates [128]. As pCR necessitates complete remission of primary tumor and lymph nodes (with the latter usually not affected by brachytherapy), approaches using external beam techniques for dose escalation seem more meaningful. Of note, none of the mentioned trials reported excessive grade 3+ late toxicity (0–11%). Therefore, further evaluation of moderate radiation dose escalation in larger trials seems to be one reasonable strategy to improve pCR rates.

**Table 6:** Prospective trials with neoadjuvant concurrent chemoradiation with radiation dose escalation or altered fractionation

Author	Year	Phase	Disease stage	n	RT dose	Conc. CHT	Adj. CHT	pCR rate	Downst	Tox gr.3+	Postop. C	late gr3+
Meade [110]	1995	po	T3/4	20	45–54 (25–30 Fx)	5-FU	dis	35%	70%^31^	10%	n.r	5%
Movsas [111]	1998	I	T3/4^41^	27	54.6–61.8 (33–39 Fx)^1^	5-FU	5-FU/LV	17%	57%^31^	37%	13%	n.r
Mohiuddin [112]	2000	po	Fixed	15	45–50 (25 Fx)	5-FU b or ci	n.s	**13%**	n.r	33%^37^	n.r	9%
				18	55–60 (30 Fx)	5-FU b or ci		**44%**				
Pfeiffer [113]	2005	I/II	Irres. or rec	18	60 (30 Fx)^2^	UFT (esc)	n.s	11%	n.r	7%	n.r	n.r
Jakobsen [114]	2006	po	T3	50	60 (30 Fx)^2^ + Br. 1 × 5	UFT	n.s	27%	n.r	10%	8%^40^	n.r
Movsas [115]	2006	II	T3/4^41^	22	61.8 (39 Fx) 3	5-FU	5-FU/LV	n.r	50%^31^	14%	14%	n.r
Freedman [116]	2007	I	T3/4 or N+	8	55 (25 Fx) 4	Cap	n.s	0%	50%^31^	38%	n.r	n.r
El-Sayed [117]	2008	II	T3/4	17	45 (25 Fx) + Br. 2 × 4	Cap + Ox	n.s	47%	n.r	n.r	n.r	0%
Jakobsen [99]	2008	II	T3/4	35	60 (30 Fx)^2^ + Br. 1 × 5	UFT + Cele	n.s	21%	n.r	6% (49%^38^)	n.r	n.r
Vestermark [118]	2008	II	Irres. or rec	52	60 (30 Fx)^2^	UFT + LV	n.s	13%	n.r	8%	n.r	n.r
Ballonoff [119]	2008	II	T3/4 or N+	8	55 (25 Fx)^4^	Cap	Cap or 5-FU/LV	38%	63%^31^	13%	n.r	n.r
Lindebjerg [120]	2009	II	T3/4	135	60 (30 Fx)^2^	5-FU/LV	n.s	19%	n.r	n.r	n.r	n.r
Marsh [121]	2010	II	T3/4 or N1	16	50.4–55.2 (42–46 Fx)^5^	Cap	Reco	18%	69%^31^	19%	n.r	n.r
Maluta [122]	2010	II	T3/4 or N+	70	60 (30 Fx)^6^	5-FU + Ox + HT	dis	24%	63%^31^	n.r	3%^40^	3%
Arbea [123]	2011	II	T3/4 or N+	100	47.5 (19–20 Fx)	Cap + Ox	CAPOX^24^	13%	n.r	n.r	5%^40^	n.r
Caravatta [124]	2011	II	T3/4 or N+	25	55 (25 Fx)^8^	Ral + Ox	Reco.^25^	32%^29^	76%^31^	8%	n.r	n.r
Barsukov [125]	2012	II	Fixed T4	64	40 (10 Fx)^9^	Cap + Ox + HT	n.s	11%	n.r	20%	n.r	n.r
Caravatta [126]	2012	I/II	T3/4 or N+	46	50.4 (28 Fx)	Ral	n.s	0%^29^	0%^32^	DLT 0%	n.r	n.r
					55 (25 Fx)^8^	Ral		25%^29^	25%^32^	DLT 13%		
					50.4 (28 Fx)	Ral + Ox		25%^29^	31%^32^	DLT 18%		
					55 (25 Fx)^8^	Ral + Ox		25%^29^	31%^32^	DLT 25%		
Li [127]	2012	II	T3N0 or N+	63	50.6 (22 Fx)^10^	Cap	dis	31%	79%^31^	n.r	7%^40^	n.r
Jakobsen [128]	2012	III	T3-4 or N+	248	50.4 (28 Fx)	UFT/LV	dis	18%	n.r	n.s. diff	8%^40^	n.r
					50.4 (28 Fx) + Br. 2 × 5	UFT/LV		18%			5%^40^	
Vestermark [129]	2012	I	Irres. or rec	18	60 (30 Fx)^2^	UFT/LV + Ox (esc)^21^	n.s	33%	Res. 83%	n.r	n.r	n.r
Mohiuddin [51, 52]	2013	II, rand	T3/4	106	55.2–60 (46–50 Fx)^11^	5-FU ci	Reco.^26^	28%	78%^31^	42%	n.r	4%
RTOG 0012					50.4–54 (28–30 Fx)	5-FU ci + Iri		28%	78%^31^	51%	n.r	8%
Zhu [130]	2014	II	T3/4 or N+	78	55 (25 Fx)^12^	Cap + Ox^22^	CAPOX	24%	n.r	n.r	n.r	n.r
Cubillo [131]	2014	po	T3 or N1	16	57.5 (23 Fx)^13^	Cap + Iri/Ox/Bev^23^	dis	50%	75%^31^	25%	n.r	n.r
Hernando [132]	2014	po	T3/4 or N+	74	57.5 (23 Fx)^13^	Cap	n.s	31%	n.r	18%	7% (gr. 4)	n.r
Appelt [133]	2015	po	T2/3 N0-1	55	60 (30 Fx)^14^ + Br. 1 × 5	UFT	Reco.^27^	78%^30^	n.r	12%	n.a	7%
Omidvari [134]	2015	II, mat	T3/4 or N+	34	45 (25 Fx) + Br. 3 × 5^15^	Cap + Ox	n.s	**29%**	n.r	6%	n.r	n.r
				102	45 (25 Fx)	Cap + Ox	n.s	**12%**		1%		
But-Hadzic [135]	2016	II	T3/4 or N+	51	46.2–48.4 (22 Fx)^16^	Cap	n.s	26%	87%^31^	4%	9%	n.r
Picardi [136]	2016	II	T4 or rec	18	55 (25 Fx)^8^	Ral + Ox	n.s	28%	89%^33^	44%	n.r	11%
Hall [137]	2016	NODA	T3/4 or N+	3298	< 45^16^	5-FU or Cap	n.s	n.r	n.r.^34^	n.r	n.r	n.r
					45^16^			**11%**	**37%**^**34**^			
					50.4^16^	w/wo Ox		**16%**	**44%**^**34**^			
					54^16^			**19%**	**49%**^**34**^			
Picardi [138]	2016	II	T3/4 or N+^42^	18	57.5 (25 Fx)^18^	Cap + Ox	n.s	25%	n.r	44%	n.r	n.r
Tey [139]	2017	II	T3/4 or N+	23	55 (25 Fx)^4^	Cap	Cap or CAPOX	35%	n.r	5%	0%	5%
Idasiak [140]	2017	II	T2N+ or T3/4	53	42 (28 Fx)^17^	5-FU b	reco.^28^	11%	n.r	8%	8%^40^	n.r
Alongi [141]	2017	po	Loc. adv	40	60 (30 Fx)^19^	Cap	n.s	18%	n.r	n.r	10%	3%
Gunther [142]	2017	II, mat	T3/4 or N+	76	52.5 (25 Fx)^20^	5-FU ci	5-FU	17%	n.r.^36^	n.s. diff	n.s. diff	n.r
				76	45 (25 Fx)	5-FU ci	5-FU	16%				

n = number of patients, RT dose: radiation dose in Gy (Fx: number of fractions), conc. CHT: concurrent chemotherapy, adj. CHT: adjuvant chemotherapy, pCR rate: percentage of patients with pathologic complete regression, downstaging: percentage of patients with major downstaging defined according to study protocol (only listed if combined T and N downstaging was reported), tox gr.3+: acute grade 3+ toxicity during chemoradiation (only listed if an overall percentage was reported), postop. c.: postoperative complications (only listed if an overall percentage was reported), late gr.3+: grade 3+ late complications (only listed if an overall percentage was reported), NODA: National Oncology Data Alliance database TM, po: prospective observational, rand.: randomized, mat.: with matched pair arm, cm: centimeter, irres.: irresectable, rec.: recurrent, 5-FU: 5-fluorouracil, b: bolus, ci: contineous infusion, UFT: uracil tegafur, esc: dose escalated, Cap: capecitabine, Ox: oxaliplatin, Cele: celecoxib, LV: leucovorine, HT: hyperthermia, Rali: ralitrexed, Iri: irinotecan, Bev: bevacizumab, w/wo: with or without, dis.: at the discretion of the treating physician, n.s.: not specified, reco.: recommended, CAPOX: combination regimen including capecitabine and oxaliplatin, n.r.: not reported, DLT: dose limiting toxicity, n.s. diff.: not significantly different, n.a.: not applicable, seq.: sequential, bid: 2 fractions per day, conco: concomittant, SIB: simultaneous integrated boost, Br.: brachytherapy boost, 1: 45 Gy in 25 Fx to pelvis, seq. boost 9.6–16.8 Gy (1.2 Gy bid), 2: 48.6 Gy in 27 Fx to pelvis with conco. boost 5.4 Gy /27 Fx + seq. boost 6 Gy (3 Fx), 3: 45 Gy in 25 Fx to pelvis, seq. boost 16.8 Gy (1.2 Gy bid), 4: 45 Gy in 25 Fx to pelvis, SIB to 55 Gy, 5: 50.4 Gy (1.2 Gy bid) for T3, 54 Gy (1.2 Gy bid) for T4, 6: 50 Gy in 25 Frx to pelvis, seq. boost 10 Gy/5 Fx, 8: 45 Gy in 25 fx to pelvis, conco boost 10 Gy/25 Fx, 9: 3 fx per week, 10: 41.8 Gy in 22 Fx to pelvis, SIB to 50.6 Gy, 11: 55.2–60 Gy (1.2 Gy bid), 12: 50 Gy in 25 Fx to pelvis, conco. boost 5 Gy /5 Fx, 13: 46 Gy in 23 Fx to pelvis, SIB 57.5 Gy, 14: 50 Gy in 30 Fx to pelvis, SIB to 60 Gy, 15: Low-dose-rate Brachytherapy, 16: 41.8 Gy in 22 Fx to pelvis, SIB 46.2 for T3, 48.4 Gy for T4, 16: conventional fractionation (altered fractionation excluded from analysis), 17: 42 Gy (1.5 Gy bid), 18: 45 Gy in 25 Fx to pelvis, SIB to 57.5 Gy, 19: 54 Gy in 30 Fx to pelvis, SIB to 60 Gy, 20: 45 Gy in 25 Fx to pelvis, conco. boost last week 7.5 Gy/5Fx, 21: one additional cycle of chemotherapy prior to and after chemoradiation prior to surgery, 22: one cycle of chemotherapy after chemoradiation prior to surgery, 23: Cap. combined with Iri or Ox or Bev according to KRAS-, BRAF-, ERCC-1, Topo-1-Status, 24: in patients with cN+, pN+ or poor response, 25: if ypN+, 26: 6: adjuvant chemotherapy was recommended for all patients with residual disease, in patients without residual disease Chemotherapy was at the discretion of the treating physician, 27: chemotherapy was recommended after surgery in case of T4, R1, EVSI, Pn1, N+ or G3, 28: chemotherapy was recommended if N+, 29: pCR defined as ypT0, 30: clinical complete response, 31: downstaging defined as reduction of p stage compared to c stage, 32: downstaging defined as ypT0-mic, 33: downstaging defined as achieving secondary resectability, 34: downstaging defined as ypT0-2N0, 36: significantly increased rate of T downstaging by RT dose escalation, 37: both arms together, 38: treatment with celecoxib terminated due to rash although all grade 1–2, 39: 18% vs 4% for 19 fx vs 20 fx arm, 40: re-operation rate, 41: only T3 < 4 cm, 42: recurrent tumors eligible

All of the mentioned strategies aimed at enhancing either the chemo- or the radiation part during concurrent chemoradiation and therefore allowed only moderate escalations due to concerns of toxicity. However, dose-intense combination chemotherapy regimens alone may result in considerable rates of downstaging and pCR rates as indicated by several studies [143]. Therefore, it seems reasonable to combine chemoradiation with sequential dose-intense combination chemotherapy in the neoadjuvant setting to improve pCR rates (known as TNT). Similarly to other diseases, this should result also in enhanced treatment compliance compared to adjuvant chemotherapy and further targets the unsolved problem of high distant metastases rates in rectal cancer by early initiation of systemic treatment. Several trials have already reported encouraging results using different schedules of sequential radio(chemo)therapy and combination chemotherapy ([20, 24, 25, 64, 88, 144–162], Table 7). Reported pCR rates ranged from 14 to 37% (weighted average 21%) in the TNT arms compared to 11–25% (weighted average 14%) in the standard chemoradiation arms of the comparative studies, indicating the superiority of the TNT approach. Moreover, the largest randomized trials observed significant benefits in terms of disease-free in the TNT arms mainly attributed to a reduction of distant failures [24, 156, 160–162], although using slightly different approaches. The Timing of Rectal Cancer Response to Chemoradiation Consortium in the United States [24, 156] performed a sequential cohort phase II study including 259 patients with T3/4 or nodal positive patients. All received upfront long-course chemoradiation (50 Gy with 5-FU c.i.) and were sequentially scheduled to receive either no or 2–6 cycles of mFOLFOX6 consolidation chemotherapy prior to surgery [24]. Chemotherapy was completed postoperatively aiming at similar total numbers of chemotherapy cycles for all four arms. The pCR rate significantly increased with the number of consolidation chemotherapy cycles from 18% (none) to 38% (6 cycles) [24]. Three-year DFS rates were also significantly increased for all TNT arms compared to the standard arm, although it has to be noted that the mean number of total chemotherapy cycles was lower in the standard arm [156]. The Polish group [157] conducted a phase III trial randomizing 515 patients with fixed T3 or T4 tumors to long-course chemoradiation (50, 4 Gy with 5-FU, leucovorin and, partly, oxalipatin) or to short-course radiation (5 × 5 Gy) followed by 3 cycles of consolidation chemotherapy with FOLFOX prior to surgery [157]. They observed no significant differences in R0-resections rates (primary endpoint), pCR rates or DFS. The significant OS benefit at 3 years (73% vs 65%) [157] disappeared with longer-follow-up [20]. The RAPIDO group [160, 161] used a similar approach randomizing 911 patients with high risk rectal cancer (defined as cT4, cN2, EMVI+, MRF+ or positive lateral nodes) to either long-course chemoradiation (50,4 Gy + Capecitabine) or 5 × 5 Gy followed by six cycles of consolidation chemotherapy with CAPOX or nine cycles of FOLFOX [160]. They found significantly improved pCR rates (28% vs 14%) favoring the TNT arm, which came at the cost of significantly increased acute grade 3+ toxicity (48% vs 25%) [160, 161]. Moreover, they described a significant benefit for the TNT arm in terms of disease-related treatment-failure (24% vs 30%) [161]. Finally, the French Group [162] tested TNT using induction chemotherapy in 461 patients with T3/T4 lesions. The patients either received long-course chemoradiation (50 Gy + Capecitabine) followed by surgery and adjuvant chemotherapy (12 × mFOLFOX6 or 8 × Capox) or induction chemotherapy with 6 cycles of mFOLFIRINOX followed by chemoradiation, surgery and less intensive adjuvant chemotherapy (6x mFOLFOX6 or 4x CAPOX) [162]. Similar to the RAPIDO trial, they described significantly improved pCR rates (28% vs 12%) and 3-year-DFS rates (76% vs 69%) for the TNT arm [162].

**Table 7:** Selected trials evaluating total neoadjuvant therapy approaches

Author	Year	Phase	Stage	n	Ind. CHT	RT dose	Conc. CHT	Cons. CHT	Adj. CHT	pCR (%)	Downst	3y-DFS	3y-OS	Tox gr.3+	Pop. c
Chau [144]	2006	II	High risk^1^	105	4xCAPOX	54	Cap	None	4xCap	20	n.r	68%	83%	n.r	n.r
Chua [145]	2010														
Koeberle [146]	2008	II	T3/4	60	1xXELOX	45	Cap/Ox	None	dis	23	n.r	n.r	n.r	n.r	n.r
Zampino [147]	2009	II	T3/4 or N+	51	None	50,4	Cap	2xCap	Cap or CAPOX 6	18	30%^9^	85%^12^	n.r	n.r	n.r
Fernandez [148]	2010	II	T3/4 or N+	108	None	50,4	Cap/Ox	None	4xCAPOX	13	53%^10^	64%^12^	78%^12^	**CRT 29%/adj. 54%**	7%^21^
Fernandez [149]	2015				4xCAPOX	50,4	Cap/Ox	None	None	14	35%^10^	62%^12^	75%^12^	**CRT 23%/ind. 19%**	8%^21^
Nogue [150]	2011	II	T3/4 or N+^2^	47	4xXELOX/Bev	50,4	Cap/Bev	None	rec	36	69%^10^	n.r	n.r	n.r	24%^21^
Chiorean [151]	2012	II	T3/4 or N+	22	2xCap/Iri	50,4	Cap	None	rec	33	n.r	76%	n.r	n.r	n.r
Di Petrillo [64]	2012	II	T3/4 or N+	26	2xmFOLFOX6/Bev	50,4	5-FU ci/Ox/Bev	None	6xmFOLFOX6/Bev	20	n.r	80%^13^	95%^13^	n.r	n.r
Marechal [152]	2012	II	T3/4	57	None	45	5-FU ci	None	dis	28	35%^11^	n.r	n.r	CRT 7%	n.r
			Or T2N+		2xmFOLFOX6	45	5-FU ci	None	dis	25	32%^11^	n.r	n.r	CRT 7%/ind. 25%	n.r
Dewdney [88]	2012	II, r	High risk^3^	165	4xCAPOX	50,4	Cap	None	CAPOX	15	n.r	Not sig	Not sig	n.r	n.r
Expert-C					4xCAPOX/Cet	50,4	Cap/Cet	None	CAPOX/Cet	18	n.r	Diff	Diff	n.r	n.r
Zhu [153]	2013	II	T3/4 or N+	42	None	44 5	Cap/Ox	1xCap	6-8xXELOX	16	n.r	57%	66%	n.r	n.r
Gao [154]	2014	II	T3/4 or N+	51	1xXELOX	50	Cap/Ox	1xXELOX	4xXELOX	42	n.r	n.r	n.r	n.r	11%
Borg [155]	2014	II, r	T3	91	6xFOLFOX/Bev	45	5-FU ci/Bev	None	n.s	**24**	66%^9^	n.r	n.r	50%	22%
					None	45	5-FU ci/Bev	None	n.s	**11**	55%^9^	n.r	n.r	20%	22%
		II	T3/4 or N+	259	None	50	5-FU ci	None	8xmFOLFOX6	**18**	n.r	**50%**^**12**^	79%^12^	cons. n.a.^15,16^	9%
Garcia-A [24]	2015							2xmFOLFOX6	6xmFOLFOX6	**25**	n.r	**81%**^**12**^	92%^12^	cons. 4%^15,16^	6%
Marco [156]	2018							4xmFOLFOX6	4xmFOLFOX6	**30**	n.r	**86%**^**12**^	88%^12^	cons. 18%^15,16^	4%
								6xmFOLFOX6	2xmFOLFOX6	**38**	n.r	**76%**^**12**^	84%^12^	cons. 35%^15,16^	9%
Bujko [157]	2016	III	fix. T3	515	None	5 × 5	None	3xFOLFOX	dis	16	n.r	43%^26^	49%^26^	24%	n.r
Cisel [20]			or T4		None	50.4	5-FU/LV/Ox	None	dis	12	n.r	41%^26^	49%^26^	24%	n.r
Perez [158]	2017	II	T3/4 or N+	39	8xmFOLFOX6	50.4	Cap	None	n.s	33	56%^10^	80%^13^	n.r	n.r	13%
Cercek [159]	2018	re	T3/4 or N+	308	5xCAPOX^19^	50–50,4	5-FU ci or Cap	None	None	18^7^	n.r	n.r	n.r	n.r	n.r
				320	None	50–50,4	5-FU ci or Cap	None	CAPOX^20^	17^8^	n.r	n.r	n.r	n.r	n.r
Fokas [25]	2019	II, r	T3/4 or N+	306	3 × 5-FU/LV/Ox	50,4	5-FU ci/Ox	None	None	17	n.r	n.r	n.r	CRT 37%/CHT 22%	17%
ARO-2012					None	50.4	5-FU ci/Ox	3 × 5-FU/LV/Ox	None	25	n.r	n.r	n.r	CRT 27%/CHT 22%	16%
van der Valk [160]	2020	III	High risk^4^	911	None	50.4	Cap	None	Opt.^22^	**14**	n.r	**30%**^**14**^	89%	**25%**^**17,23**^	15%
Hospers [161] RAPIDO					None	5 × 5	None	6xCAPOX^18^	Opt.^22^	**28**	n.r	**24%**^**14**^	89%	**48%**^**17,23**^	14%
Conroy [162]	2020	III	T3/4	461	None	50,4	Cap	None	8xCap^24^	**12**	n.r	**69%**	88%	n.r	n.r
PRODIGE 23					6 × mFOLFIRINOX	50,4	Cap	None	4xCap^25^	**28**	n.r	**76%**	91%	n.r	n.r

n = number of patients, ind.: induction CHT (prior to chemoradiation), RT dose: radiation dose in Gy, conc. CHT: chemotherapy concurrent to radiation, cons. CHT: consolidation chemotherapy (after chemoradiation prior to surgery), adj. CHT: adjuvant chemotherapy after surgery, pCR rate: percentage of patients with pathologic complete regression, downstaging: percentage of patients with major downstaging defined according to study protocol (only listed if combined T and N downstaging was reported), 3y-DFS: 3-year disease free survival, 3y-OS: 3-year overall survival, grade 3+ tox: acute grade 3+ toxicity during chemotherapy and/or chemoradiation (only listed if an overall percentage was reported), pop.c.: postoperative complications grade 3+ (only listed if an overall percentage was reported), r.: randomized, re.: retrospective, fix.: fixed tumors, CAPOX: combination regimen including capecitabine and oxaliplatin, XELOX: combination regimen including capecitabine and oxaliplatin, Bev: Bevacizumab, Cap: Capecitabine, Iri: irinotecan, mFOLFOX6: combination regimen including 5-FU, leucovorine and oxaliplatin, Cet: Cetuximab, 5-FU: 5-fluorouracil, LV: leucovorine, Ox: Oxaliplatin, ci: contineous infusion, b: bolus, FOLFOX: combination regimen including 5-FU, leucovorine and oxaliplatin, dis.: at the discretion of the treating physician, rec.: recommended, n.s.: not specified, opt.: optional, n.r.: not reported, no. sig. diff.: no significant difference between arms, CRT: chemoradiation, n.a.: not applicable, 1: T3c or T4 or MRF+ or N2 orT3 at levator level,: T3 only if distal located or < 2 mm from MRF, 3: T3c or T4 or MRF+ or EVSI or tumor at levator level, 4: T4 or N2 or EVSI or MRF+ or positive lateral nodes, 5: in 20 fractions, 6: if pCR none, if N0 Cap., if N+ CAPOX, 7: 24% of the patients chose non-oprative management due to clinical complete remission, 8: 8% of the patients chose non-oprative management due to clinical complete remission, 9: downstaging defined as lower p vs c stage, 10: downstaging defined as ypstage 0–1, 11: downstaging defined as ypT0-1N0, 12: 5-year rate, 13: estimated from curve, 14: disease-related treatment failure (DrTF), 15: CRT toxicity reported for all groups together (28%), did not significantly differ between the groups, 16: toxicities for adjuvant CHT not reported, significantly less patients received adjuvant chemotherapy in group 1, 17: toxicities reported only for preoperative part of treatment, 18: or 9 × FOLFOX, 19: or 8 × mFOLFOX6, 20 or mFOLFOX6, 21: re-operation rate, 22: predifined strategy by center, either none or 8 × CAPOX or 12 × FOLFOX, 23: 35% for adjuvant chemotherapy, 24: or 12 × mFOLFOX (predefined choice by center), 25: or 6xmFOLFOX6 (predefined choice by center), 26: 8-year rate

Regarding the timing of chemoradiation and chemotherapy, both possible approaches (induction or consolidation chemotherapy) reached comparable results in terms of pCR rates and survival. The German CAO/ARO/AIO-12 trial [25], which directly compared induction and consolidation chemotherapy strictly using the same schedules during chemotherapy as well as chemoradiation in both arms, and thus achieving the same time interval from treatment start to surgery, found a non-significant but distinct difference in pCR rates favoring the consolidation arm (17% vs 25%). This might be explained by the longer time interval from chemoradiation to surgery, although the randomized GRECCAR-6 trial (without consolidation chemotherapy) was not able to confirm such an association [163].

In summary, several strategies to improve the pCR rate by neoadjuvant treatment intensification currently exist with the TNT approach probably being the most promising as it targets not only pCR rate but also seems to reduce distant failure rates with improved treatment compliance and acceptable toxicity. Although the most recent phase III trials (RAPIDO, PRODIGE 23) have been published only in abstract form so far, TNT will probably be the new standard of care for high-risk rectal cancer patients with the detailed treatment algorithm regarding to different subgroups yet to be defined.

Combination of the TNT approach with intensification of the concurrent treatment phase by moderate radiation dose escalation might be a reasonable further direction of research especially if non-operative management strategies (as addressed in the following part) are taken into account.

## Organ-preserving surgery and non-operative management

### Organ-preserving surgery

Local excision (LE, e.g. in the form of transanal endoscopic microsurgery, TEM) was initially tested as an organ preserving surgical alternative in prospective, non-randomized phase 2 studies for cT1/2 and early cT3 tumors in the lower third of the rectum after RCT with the aim of avoiding abdominoperineal resection with permanent stoma or resection with coloanal anastomosis and its morbidities while maintaining the same oncological safety. Meanwhile, results from two prospective randomized studies are available. An Italian study randomized 100 patients with distal cT2N0 tumors after neoadjuvant RCT between TEM and laparoscopic TME [164]. In both arms the R0 resection rate was 100%. Conversion to radical surgery was not performed in either patient in the TEM arm. Patients operated with TEM had a significantly shortened operation time and less blood loss, but postoperative complications did not differ significantly in both groups. With a follow-up of 9.6 years there were 4 local recurrences and 2 distant metastases after TEM, 3 local recurrences and 2 distant metastases after TME. Data on quality of life, functional outcome and late morbidity were not reported.

The French GRECCAR 2 trial was a prospective, multicenter phase 3 trial that randomized patients with cT2/3 N0-1 tumors up to 8 cm from the anal verge and with good response (residual tumor < 2 cm) 6–8 weeks after RCT into an LE versus TME group [165]. In the LE group, TME completion surgery was performed in case of ypT2-3 or R1. Of 186 patients included, 148 (80%) showed good response and 145 were randomized. In the LE arm, 26/74 patients (35%) required TME completion surgery. The primary endpoint was a combination of events: death, recurrence, surgical complication rate grade 3–4, and severe adverse events at 2 years (anal incontinence, impotence, definite colostoma). One or more of these events were observed in the intention-to-treat analysis after 2 years in 41/73 patients (56%) in the LE group and in 33/69 patients (48%) in the TME group (*p* = 0.43). There were no significant differences in the individual components of the combined endpoint between the two randomization groups, with the subgroup of patients with TME completion surgery after LE performing particularly poorly in the cumulative score of surgical complication rates and adverse events after 2 years (29% after LE, 38% after TME, 78% after LE plus TME). With a follow-up of 60 months both treatment groups showed no significant differences in oncological 5-year endpoints (LE arm vs. TME arm: local recurrence rate 7% vs. 7%, distant metastasis rate 18% vs. 19%, disease-free survival 70% vs. 72%, overall survival 84% vs. 82%).

Overall, the role of LE/TEM after RCT is not sufficiently clarified. Prognostically unfavorable findings after LE (R1, ypT2/3, ypN+) usually require TME completion surgery, which may be associated with significantly higher surgical complication rates, poorer long-term functional outcome and a higher rate of definitive colostomas than primary TME. Thus, the concept of neoadjuvant RCT followed by LE and possibly followed by TME completion surgery represents a potentially significant overtreatment especially for patients with early rectal cancer. Further studies will clarify which histopathological findings after LE require TME completion surgery (e.g. does every ypT2 after LE require TME?). Finally, it remains to be clarified how functional endpoints and aspects of quality of life/morbidity after RCT and LE compare to primary radical surgery.

### Nonoperative management following neoadjuvant standard chemoradiotherapy

Investigators from the University of Sao Paulo were the first to pioneer the selective nonoperative management (NOM) approach for patients with potentially resectable rectal distal cancer who experience a clinically complete response (cCR) to chemoradiotherapy (CRT) [166]. In early reports, Habr-Gama et al. described the outcomes of 361 patients with cT2-4 and or cN+ distal rectal cancer treated with standard neoadjuvant CRT (50.4 Gy plus 5-FU/folinic acid) and assessed for response 8 weeks after completion of CRT with clinical, endoscopic, and radiologic studies. Patients with initial cCR (n = 122, 34%) underwent a strict watch-and-wait (W&W) strategy with monthly examinations for the first year; 23 of these 112 patients (19%) developed local tumor regrowth within 12 months. Only patients without any local regrowth within the first year of follow-up were considered to have a “sustained cCR”. A total of 99 of 361 (27.4%) patients met the criteria for sustained cCR and had a mean follow-up of 60 months, during which 5 patients developed endoluminal (all salvaged), 7 distant, and 1 combined recurrences [167].

Maas et al. aimed to replicate the results from Sao Paulo, evaluating also the role of modern MRI techniques in the selection and follow-up of patients [168]. Re-staging was performed 6–8 weeks after completion of standard CRT (50.4 Gy, concurrent capecitabine) for clinically T3-4 and/or N+ rectal cancer patients by use of digital rectal examination, high-resolution MRI and endoscopy plus biopsies. If these examinations indicated no residual tumor or residual fibrosis only, patients were eligible for NOM combined with intensive follow-up: 21 of the 192 (11%) patients had evidence of cCR. With a median follow-up of 25 months, only one patient developed a local recurrence (successfully treated with salvage surgery), 20 patients are alive without disease. Patients with cCR included in a wait-and-see policy did at least as good as a control group of 20 patients with a pCR after radical surgery, but had less toxicity and better short-term bowel function. In a more recent update of this strategy, including 100 patients with cCR and a median follow-up of 41 months, local regrowth occurred in 15 patients (12 luminal, 3 nodal), all salvageable, with a 3-year local regrowth-free survival of 85%, and a 3 year overall survival of 97%. Continence after watch-and-wait based on the Vaizey incontinence score was excellent [169]. A retrospective analysis from the Netherlands with 41 W&W patients who were matched with 41 patients who had undergone standard CRT and TME reports better functional results with regard to continence, defecation, micturition, and sexuality as well as a globally better quality of life in the W&W Group [170].

Meanwhile, several population-based data collections on the W&W strategy are available. The British "OnCoRe" project reports on 129 patients who achieved cCR following standard CRT and chose a W&W strategy. With a median follow-up of 33 months, 44 patients (34%) developed local regrowth, which could be treated with curative salvage surgery in 41 patients [171]. A propensity score cohort analysis showed no significant difference for DFS and OS compared to a cohort of patients matched by T-category, age and performance status. Colostomy-free survival was significantly better in the W&W Group. The largest population-based data collection to date, the International Watch&Wait Database (IWWD), reports on 880 patients with cCR after neoadjuvant CRT from 47 institutions and 15 countries [172]: with a median follow-up of 3.3 years local regrowth was of 25.2% after 2 years; 95% of these recurrences were localised in the rectal wall, and 88% were diagnosed within the first 2 years. The rate of distant metastases was low at 8%, disease-specific survival and overall survival was favourable at 94% and 85% after 5 years. Systematic reviews and meta-analyses of retro- and prospective studies on the W&W strategy describe pooled local regrowth rates of 15.7% after 2 years and 21.6% after 3 years [173, 174]. The incidence of distant metastases is consistently low in these meta-analyses (6.8% after 3 years in [174]).

### Can organ preservation by NOM be further optimized?

Habr-Gama et al. reported the results of a more intense CRT regimen of 54 Gy in 30 fractions with 3 concurrent cycles of 5-FU/folinic acid every 21 days, followed by 3 further cycles of consolidation chemotherapy before response assessment 9 weeks after completion of CRT: initial cCR in 70 patients with T2-3 distal rectal cancer was 68%, and sustained cCR at 1 year of follow-up 57% [175]. Another prospective watch-and-wait approach from Denmark evaluated patients with low-lying (< 6 cm from anal verge) cT2-3, cN0-1 rectal cancer [133]. Patients were treated with an increased radiation dose (60 Gy in 30 fractions with an additional 5 Gy endorectal brachytherapy boost) and concurrent oral tegafur-uracil. Response was assessed 6 weeks after CRT by endoscopy/biopsy and MRI, and complete responders were prospectively observed. A total of 40 out of 51 eligible patients (78%) met the criteria of cCR. With a median follow-up of 24 months, the 1- and 2-year cumulative incidence of local regrowth for these 40 patients were 15% and 26%, respectively. All these patients were successfully salvaged without additional recurrences.

An emerging body of data suggests that—reminiscent to anal cancer treatment—the response to CRT in patients with rectal cancer is time-dependent, and maximal tumor regression may well take longer than the standard 6–8 weeks, especially if consolidation chemotherapy is used following CRT [24, 25]. As mentioned above, The Timing of Rectal Cancer Response to Chemoradiation Consortium in the United States conducted a prospective phase 2 trial comparing preoperative CRT alone with CRT followed by increasing numbers of consolidation chemotherapy cycles (2–6) and thus increased time intervals from CRT to surgery (11, 15 and 19 weeks) [24]. The pCR rate of patients treated in study group 1 was 18% compared with 25%, 30%, and 38%, respectively, for study groups 2–4 without an apparent increase in surgical complications. Based on these results, the US organ preservation of rectal adenocarcinoma (OPRA) randomized phase 2 trial tested the feasibility of using CRT and either induction or consolidation chemotherapy for patients with MRI-staged T2-3, N0 or Tany N1-2 rectal cancer. First results, presented at ASCO 2020, showed an impressive rate of 58% organ preservation at 3 years for CRT followed by 4 months of FOLFOX/CAPOX versus 43% organ preservation for induction chemotherapy followed by CRT (*p* = 0.01) with no differences in DFS (77 versus 78%, respectively) [176].

### Caveats of the NOM

Despite these promising data, further prospective studies with sufficient patient numbers and follow-up are needed to better assess the risk/benefit ratio for patients who choose NOM. There have been concerns that individual patients might be disadvantaged by the omission of surgery after being diagnosed with a cCR. First, patients with initially resectable tumors might develop irresectable regrowth or lesions that require abdominoperineal resection while low anterior resection would have been sufficient in the first place. The second concern is the development of distant metastases that do no longer allow curative treatment. While patients need to be informed about the still experimental character of NOM, the current literature suggest the oncological safety of this approach. Of note, clinical examination, endoscopy and MRI to identify patients with cCR and to detect local regrowth during close follow-up require a high level of expertise and should be restricted to centers with special experience in multimodal diagnosis and therapy of rectal cancer, including NOM.

## Conclusion

The role of radiation therapy and the further direction of treatment optimization have changed dramatically in the recent decade. The initial problem of high local recurrences rates after surgery, which prompted the introduction of radiation therapy into the treatment algorithm, seems to be solved for the vast majority of patients. Due to improvements in imaging and surgical technique we are now facing the contrast of evaluating the omission of radiation therapy in patients with low risk for local recurrence or the intensification of neoadjuvant (radiation therapy containing) approaches aiming at the improvement of oncological outcome in high risk patients or even attempting rates of complete remissions which allow non-operative organ-preserving approaches. Several strategies for treatment intensification exist, with the TNT approach seeming the most promising option due to its improvements not only in pCR and downstaging rates, but also in reduction of distant failures without distinct increases in treatment-related toxicity compared to standard chemoradiation or SCRT with adjuvant chemotherapy. Limited (organ-preserving) surgery in patients with good response to neoadjuvant CRT seems possible in selected patients but its role needs to be clarified in further trials. Preliminary results do further suggest, that organ-preserving non-operative approaches in patients with clinical complete responses to neoadjuvant therapy are safe if proper selection and meticulous follow-up examinations are performed. Future directions may include the development of tools for a more precise identification of suitable subgroups and/or the prediction of response as well as further treatment intensification by combining TNT approaches with intensified concurrent chemoradiation approaches.

## Data Availability

Not applicable.
